# Progress in antiandrogen design targeting hormone binding pocket to circumvent mutation based resistance

**DOI:** 10.3389/fphar.2015.00057

**Published:** 2015-03-24

**Authors:** Xiaohong Tian, Yang He, Jinming Zhou

**Affiliations:** ^1^Lady Davis Institute, Jewish General Hospital, Mcgill UniversityMontreal, QC, Canada; ^2^Immunology, Institute of Medicinal Biotechnology Chinese Academy of Medical ScienceBeijing, China

**Keywords:** androgen receptor, antiandrogen, drug resistance, mutation, rational drug design

## Abstract

Androgen receptor (AR) plays a critical role in the development and progression of prostate cancer (PCa). Current clinically used antiandrogens such as flutamide, bicalutamide, and newly approved enzalutamide mainly target the hormone binding pocket (HBP) of AR. However, over time, drug resistance invariably develops and switches these antiandrogens from antagonist to agonist of the AR. Accumulated evidence indicates that AR mutation is an important cause for the drug resistance. This review will give an overview of the mutation based resistance of the current clinically used antiandrogens and the rational drug design to overcome the resistance, provides a promising strategy for the development of the new generation of antiandrogens targeting HBP.

## Introduction

Prostate cancer (PCa) is one of the most common cancer and the second leading cause of cancer death in men in the western countries (Jemal et al., 2011). AR, a member of nuclear receptor family that is activated by binding of androgens (Roy et al., 1999), plays an important role in promoting the development of PCa (Dong et al., 2005). Moreover, it has been commonly agreed that AR expression and signaling remains intact as the disease evolves from androgen-sensitive cancer to castration-resistant prostate cancer (CRPC) which are still dependent on AR signaling axis (Jenster, 1999; Taplin, 2007). Thus, AR has become the most important therapeutic target for the treatment of PCa (Aragon-Ching, 2014; Carver, 2014; Culig, 2014).

Through blocking AR signaling, androgen-deprivation therapy (ADT) (via surgical or chemical castration) has been remaining the mainstay for the treatment of advanced PCa since 1940s. (Ruckle and Oesterling, 1993; Lubeck et al., 2001; Ryan and Small, 2006; Cannata et al., 2012). Normally, ADT reduces 95% of testosterone levels. However the androgen stimulus is still persisting as a result of circulating androgens produced by intracrine steroidogenesis (Montgomery et al., 2008). Thus, by using antiandrogens as an adjuvant treatment of ATD to block the intracrine steroidogenesis, may eventually delay or prevent the progression to CRPC. AR antiandrogens prevent androgens from carrying out their biological activity by directly binding and blocking the AR LBD, or by inducing repressive activity (Maeda and Usami, 2002; Gillatt, 2006). Current clinically used antiandrogens such as flutamide (Goldspiel and Kohler, 1990), bicalutamide (Blackledge et al., 1997), and newly approved enzalutamide (Semenas et al., 2013) mainly target the HBP of the AR LBD. These approved antiandrogens have greatly improved the survival and life quality of the PCa patients. However, after the initially effective response, most tumors progress to CRPC under the treatment of antiandrogens, and no curative therapy is available nowadays.

It has been wildly accepted that the acquired AR mutation is an important cause for the drug resistance of PCa toward antiandrogens. For example, T877A mutant would turn flutamide into AR agonist (Bohl et al., 2005a), and W741C would turn bicalutamide into AR agonist. (Bohl et al., 2005b). Therefore, the development of novel antiandrogens to circumvent the mutation based resistance is highly demanded. Fortunately, up to now, several effective strategies, especially the rational drug design, have been applied in the design of the antiandrogens targeting HBP of the AR, and several promising agents have been obtained (Trendel, 2013). This review will give an overview of the mutation based resistance of the current clinically used antiandrogens and the rational drug design to overcome the resistance, which provides a promising strategy for the development of the new generation of antiandrogens targeting the AR HBP.

## Structure and function of AR

**Figure 1:**
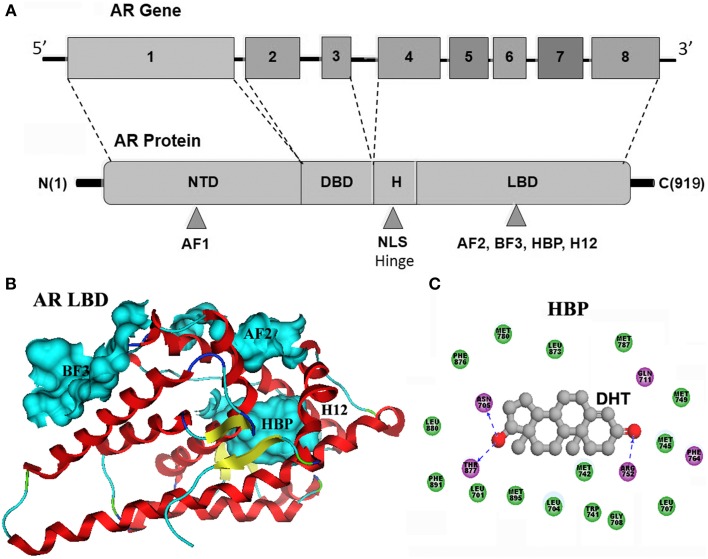
**(A)** AR gene consists of 8 exons encoding the androgen receptor with a gene product of typical size of 919 amino acids. AR is comprised of an N-terminal domain (NTD), a central DNA binding domain (DBD), a short-hinge region, and a C-terminal LBD. **(B)** LBD comprises a 12 helical structure that encloses a central hormone binding pocket (HBP), a second activation function domain (AF2) that is located at the carboxy-terminal end of the LBD, and a recently discovered binding site, Binding function 3 (BF3). The adopted conformation of H12 are unambiguously associated with the molecular mechanism of action of ligands bound to the HBP. **(C)** As shown in compex structure of dihydrotestosterone (DHT) and AR-LBD, the AR HBP is primarily composed of hydrophobic residues (green ball) that can form strong nonpolar interactions with DHT. The protein-ligand anchoring can be additionally stabilized by a network of hydrogen bonds (blue dashed line) involving R752, Q711, N705, and T877 polar residues.

As a member of nuclear receptor family, AR possesses a modular organization characteristic to all of the nuclear receptors. There is only one AR gene identified in human so far, consisting of 8 exons encoding the AR with a typical size of 919 amino acids (Werner et al., 2006; Gao, 2010). AR is comprised of an N-terminal domain (NTD), a central DNA binding domain (DBD), a short-hinge region, and a C-terminal LBD (Figure 1A). Among these domains, the NTD, as an intrinsically disordered region, is the least conserved domain in AR, which contains a transcription activation domain: activation function 1 (AF1) that regulates gene transcription in a ligand-independent fashion (Yuan et al., 2001). The adjacent DBD is the most conserved domain, composed of two cysteine-rich zinc-finger motifs, mediating AR binding to recognition elements of specific genes in DNA. The hinge region bridges between the DBD and the LBD and harbors a nuclear localization signal (NLS). The C-terminal LBD (Figure 1B) comprises a 12 helical structure that encloses a central hormone binding pocket (HBP), a second activation function domain (AF2) that is located at the carboxy-terminal end of the LBD and mediates ligand dependent transactivation, and a recently discovered binding site, Binding function 3 (BF3). Helix 12 (H12) is the most flexible part of AR, and conformational changes of H12 are unambiguously associated with the molecular mechanism of action of ligands bound to the HBP (Caboni and Lloyd, 2013). The HBP is primarily composed of hydrophobic residues that can form strong nonpolar interactions with ligands. The protein-ligand anchoring can be additionally stabilized by a network of hydrogen bonds involving polar residues such as R752, Q711, N705, and T877 (Figure 1C).

Androgen exerts its biological effects through binding the HBP of AR. Upon binding, H12 is repositioned to cover the HBP, triggering agonist-induced conformational change in the LBD, results in the formation of AF2. The AR dissociates from heat shock proteins (HSPs), homodimerizes and translocats into the nucleus where it binds ARE sites of DNA, directly regulates targeting genes transcription in the presence of coactivator which binds to AF2, promoting the recruitment of RNA polymerase II, triggering the transcription process (Pratt and Toft, 1997; Heinlein and Chang, 2002; Shang et al., 2002). In addition to the classic genomic actions, the nongenomic actions of the androgens have also been observed in various tissues and characterized by the lack of immediate activation of transcription/translation processes, due to the rapidity of action that is mainly mediated by the activation of cytoplasmic and/or plasma membrane-associated receptors and downstream signaling pathways (Norman et al., 2004), which contributes to the overall effects of androgen stimulation, along with the classic genomic actions.

AR plays an important role in promoting the development of PCa (Dong et al., 2005). Accumulated evidence shows that the AR signaling still contributes to CRPC through several mechanisms, including AR protein overexpression, acquired mutations of AR which disables the antagonistic activity of antiandrogen, aberrant expression of AR co-regulators and alternative AR activation by cytokines and growth factors in the absence of androgens, and the expression of AR splice variants (ARvs) lacking LBD such as AR-V7 and ARv567. In addition, the TMPRSS-ERG fusion also plays an important role in PCa progression by disrupting the AR lineage-specific differentiation through gene rearrangements, which leads to an EZH2-mediated de-differentiation of cells. The most common one is the fusion of the 3′ region of ERG with the 5′ region of the highly AR-regulated TMPRSS2 gene. ERG rearrangements have been identified in 40–60% of PCa (Tomlins et al., 2005).

## AR acquired mutations driven drug resistance in PCa

**Figure 2:**
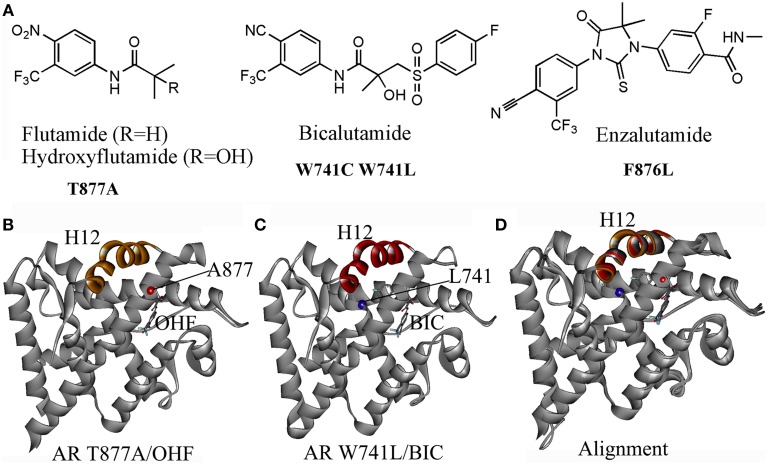
**(A)** Several acquired mutations drive drug resistance: T877A mutant turns hydroxyflutamide (OHF), an active metabolite of antiandrogen flutamide in AR agonist; W741C or W741L converts bicalutamide (BIC) into AR agonist; a novel mutant F876L was identified inducing the resistance to enzalutamide and ARN509. **(B)** The crystal structure (PDB-ID: 2ax6) of T877A LBD/ hydroxyflutamide (OHF) shows adopting a agonistic conformation; **(C)** the crystal structure (PDB-ID: 1z95) of W741L LBD/ bicalutamide (BIC) shows adopting a agonistic conformation; **(D)** A superimposition of T877A LBD/OHF and W741L LBD/ BIC structures with the LBD structure bound with DHT (PDB-ID: 1i37) by sequence conferred a perfect alignment of the H12 with the RMSD value of 0.28 and 0.48 Å, respectively, which confirmed the mutant-driven conversion from antiandrogen to AR agonist.

Current clinically used antiandrogens mainly target the HBP. There are two types of antiandrogens: the steroidal antiandrogens and the non-steroidal antiandrogens. Several steroidal anti-androgens (cyproterone acetate, megestrol acetate, and medroxyprogesterone acetate) are initially used for the androgen blockade in patients. However, severe drawbacks such as hepatotoxicity, interference with libido and potency, cardiovascular side effects and low efficacy have limited their clinical use. The later developed non-steroidal antiandrogens including the first-generation antiandrogens such as flutamide, bicalutamide, nilutamide, and the second-generation compounds: enzalutamide and ARN509. These non-steroidal antiandrogens which avoid the typical constraints of the steroidal antiandrogens, have been widely used in clinical nowadays. However, after initially effective response, approximately 50% of patients whose cancer started to grow again under the treatment of antiandrogens, the cancer has been observed to regress by simply stopping the antiandrogen, which referred to the Anti-Androgen Withdrawal Response (AAWR) (Paul and Breul, 2000; Sartor et al., 2008). It has been widely accepted that the acquired mutations of AR in PCa under the pressure of the antiandrogen is responsible for such phenomenon, which turns antiandrogens from AR antagonist to AR agonist and causing drug resistance (Figure 2A).

One particular AR mutation is the T877A in the LBD of AR, which actually results in paradoxical activation by hydroxyflutamide, an active metabolite of flutamide. Several variations of the 877 mutations have been discovered: T877S, T877C, and T877G. The T877A mutation along with H874Y mutation allows AR to be activated by cortisol. The W741C and W741L AR mutant are activated by bicalutamide. Another mutation, L701H, enhances cell proliferation upon stimulation of IL-6. Additionally, V715M, along with T877A, L701H, and H874Y, were identified in LNCaP cells treated with bisphenol A. Besides, a mutation at Q640S produces a truncated AR resulting in constitutive activation in the absence of ligand (Grasso et al., 2012; Trendel, 2013). Recently, a novel mutant F876L which turned enzalutamide and ARN509 from AR antagonist to AR agonist was identified in preclinical models and in the patients being treated with ARN509 (Joseph et al., 2013).

The mechanisms of mutant-driven conversion of antiandrogen from AR antagonist to AR agonist have been verified by the biological structural data. The crystal structure of T877A AR-LBD in complex with OHF (PDB-ID, 2ax6) demonstrates that the H12 adopts the agonistic conformation, which elucidates that the T877A mutation converts the OHF from an antagonist to an agonist (Figure 2B) (Bohl et al., 2005a). Another crystal structure (PDB-ID, 1z95) provides the structural evidence that W741L AR mutant switches bicalutamide to an agonist (Figure 2C) (Bohl et al., 2005b). A structural superimposition of these two structures with the AR-LBD structure bound with DHT conferred perfect alignments of the H12 with the RMSD (root mean square deviation) values of 0.28 and 0.48 Å, respectively, which confirmed that the mutant-driven conversion from the AR antagonist to AR agonist (Figure 2D). However, the mechanism under the antagonist-agonist conversion of ligands remains elusive.

## Rational antiandrogen design to circumvent drug resistance driven by AR acquired mutations

**Figure 3:**
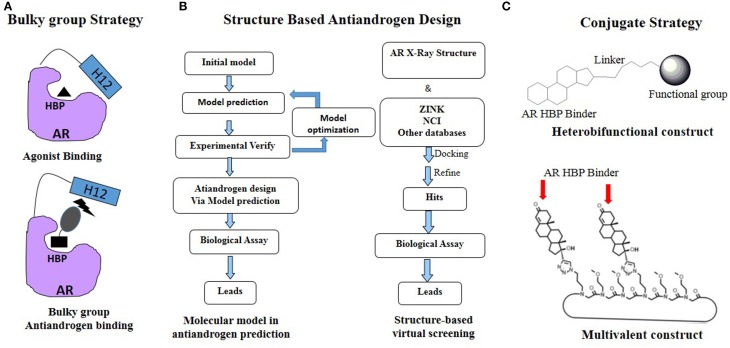
**Rational antiandrogen design strategy to combat the mutation driven drug resistance: (A) Bulky group strategy; (B) Structure based antiandrogen design; (C) Conjugate strategy**.

As mentioned previously, PCa is continually undergoing AR mutations that switch the antiandrogen from AR antagonist to AR agonist and eventually relapses to lethal CRPC. Thus the rational development of novel antiandrogens based on the AR acquired mutations driven drug resistant seems like ongoing running race and remains a big challenge (Josan and Katzenellenbogen, 2013). Up to now, several effective strategies, especially the rational drug design, have been applied in the development of the antiandrogens targeting HBP of AR (Figure 3).

### Bulky group strategy

**Figure 4:**
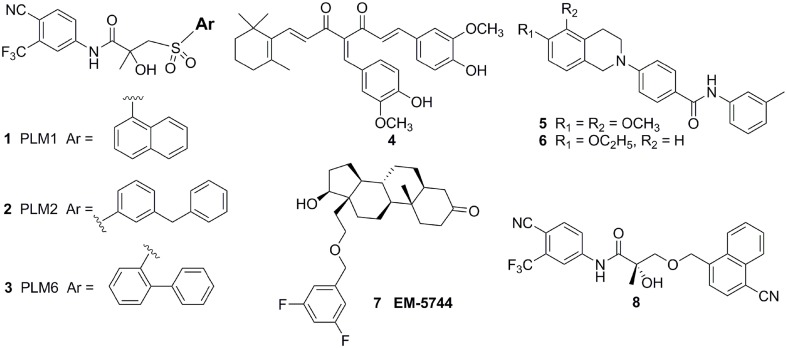
**Chemical structures of AR Antagonists and Agonists designed by using bulky group strategy**.

Considerating that the agonistic conformation of H12 is like a lid enclosing the HBP, designing compounds bearing an extended bulky arm to displace H12 (Figure 3A) is an effective strategy. For example, based on the structure of bicalutamide, several derivatives with an extended aryl sulfone core were designed and prepared. Among them, compounds **1–3** (Figure 4) showed potent antagonistic activity in all three mutations (T877A, W741L, and W741C) as well as wild-type AR (McGinley and Koh, 2007). Similarly, a series of flutamide analogs with bulky groups were also obtained and some of them circumvent the resistant mutation to their parent compound (Duke et al., 2011). Other bulky group antiandrogens with novel scaffolds were also reported. Zhou et al have developed a novel antiandrogen **4** (Figure 4) with two bulky side chains which shown low micromolar cytotoxicity in a panel of five PCa cell lines and potently suppressed DHT-induced transactivation of the WT and the T877A, W741C, and H874Y mutated ARs. Molecular modeling indicated that **4** adopts a “Y”-shape conformation and forms multiple hydrogen bonds with AR backbone in HBP (Zhou et al., 2009). Besides, via the SAR studies of the lead compound DIMN, Yang et al have synthesized a series of nicotinamides with extended linear scaffold bearing sterically bulky alkoxy groups on isoquinoline end and identified compounds **5** and **6** (Figure 4) as promising candidates of second generation antiandrogen for advanced PCa (Yang et al., 2013).

Interestingly, Endo et al have developed a series of carborane substituted antiandrogens (Fujii et al., 2005; Goto et al., 2005, 2010; Ohta et al., 2008). Carborane (dicarba-closo-dodecaborane, C2B10H12) is an icosahedral boron cluster, has a bulky spherical structure, exhibits remarkable thermal stability, and has high hydrophobicity. (Armstrong and Valliant, 2007). By replacing the hydrophobic ring such as the steroidal skeleton or the phenyl ring in hydroxyflutamide or bicalutamide by carborane cage, thus disposition of the helix-12 by using steric repulsion between the bulky carborane cage and several amino acid residues, mainly M895 and F876, in the hydrophobic pocket of the AR LBD. Several carborane containing antiandrogens showed the comparable potency to the known antiandrogens hydroxyflutamide or bicalutamide and exhibit pure AR full antagonistic property.

However, although there were a couple of successful examples, the bulky group strategy was somehow casted doubt (Duke et al., 2011). For example, several DHT-derived molecules bearing one bulky chain surprisingly turned out to be potent agonists of the AR. The complex structure of the AR LBD and one of the compounds termed EM5744 (Figure 4) (PDB-ID, 2pnu) was solved, which indicates the H12 of AR adopts the agonistic position (Cantin et al., 2007). Besides, a crystal structure (PDB-ID: 3rll) of compound **8** (Figure 4) complexed with AR (T877A) shown that receptor accommodated the added bulky groups such as phenyl to naphthyl substitution (Duke et al., 2011). The failure of the bulky ligand strategy might due to the flexibility of HBP, since it was reported that the volume of HBP would be hugely increased upon the binding of the ligands and the recruitment of coactivators (Xu et al., 2011).

### Structure based antiandrogen design targeting HBP

Recently, structural based molecular modeling has been developed to predict target mutation-induced drug resistance. Meanwhile, various structural based design strategies, including targeting protein backbone, targeting highly conserved residues and dual/multiple targeting, have been used to design novel inhibitors for combating the drug resistance (Hao et al., 2012). To date, there are 89 AR LBD structures deposited in Protein Database Bank (PDB, www.pdb.org), which facilitate the discovery and development of novel antiandrogens to combat the drug resistance by using “structure-based” drug design (Figure 3B).

**Figure 5:**
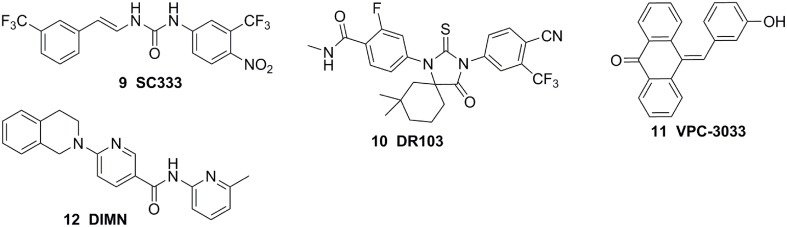
**Chemical structures of AR Antagonists based on Structure of HBP**.

The molecular modeling techniques, especially molecular dynamics, have been widely used to predict the conformational displacement of H12 when the mutant occurs. Zhou et al investigated the impact of the T877A mutation on ligand-induced helix-12 positioning by replica-exchange molecular dynamics (REMD) simulations and proposed a REMD based methodology to predict agonist/antagonist potency of a ligand. According to the simulation results, a novel flutamide derivative called SC333 (Figure 5) was designed and predicted to be a pure antagonist of the T877A mutant, which was further experimentally confirmed as a pan-antiandrogen against the wild type AR and the T877A and W741C mutated ARs (Zhou et al., 2010). In additional, Osguthorpe et al predicated that bicalutamide antagonizes AR by accessing an additional binding pocket (B-site) adjacent to the HBP via molecular dynamics, induced by displacing H12(Osguthorpe and Hagler, 2011). These molecular modeling studies based on crystal structure shed light on the mechanism of the mutant-driven antagonist/agonist conversion and provide a structural framework for the design of novel antiandrogens (Osguthorpe and Hagler, 2011). An impressive work was reported by Dr Sawyers and the collaborators recently (Balbas et al., 2013). They performed molecular dynamics simulations of antiandrogen-AR complexes and suggested the mechanism that the F876L substitution alleviates antagonism through repositioning of the coactivator recruiting H12. Based on the mechanism, a focused chemical screening was performed and three novel compounds, including the most potent compound DR103 (Figure 5), were identified effectively antagonizing AR F876L (and AR WT) to suppress the growth of PCa cells lines resistant to enzalutamide (Balbas et al., 2013).

Structure-based virtual screening is another effective way to obtain the lead compound of antiandrogens. For example, combining virtual screening and further biological assay, a lead compound, VPC-3033 (Figure 5), was identified to demonstrated strong androgen displacement potency which effectively inhibited AR transcriptional activity possess, profoundly degraded AR and significantly suppressed enzalutamide resistance PCa cells (Li et al., 2013a). Moreover, through structure-based virtual screening using the FlexX docking model, 54 candidates were selected and further screened for AR antagonism via cell-based tests. One compound, DIMN (Figure 5), showed antagonistic effect specific to AR with comparable potency to that of hydroxyflutamide and bicalutamide (Song et al., 2012). In another virtual screening work, the database was firstly filtered through a pharmacophore models built according to the structures of the reported antiandrogens, and the hits were subjected to structure-based docking evaluations using an AR homology model and cross-docked into the variant AR crystal structure bound to bicalutamide using the Surflex suite. A series of structural distinct competitive AR antagonists were obtained, which belonged to six chemotypes. Among these compounds, chemotype A compounds functioned as AR antagonists *in vivo* in normal male mice and suppressed AR activity and tumor cell proliferation in human CRPC xenografts (Shen et al., 2012). In addition, the molecular docking was also applied to establish the binding mode of the antiandrogens in HBP of the AR LBD, which would be beneficial to further optimization of the lead compounds (Zhou et al., 2009; Pepe et al., 2013; Guerrini et al., 2014).

### Steroid or non-steroid conjugates

**Figure 6:**
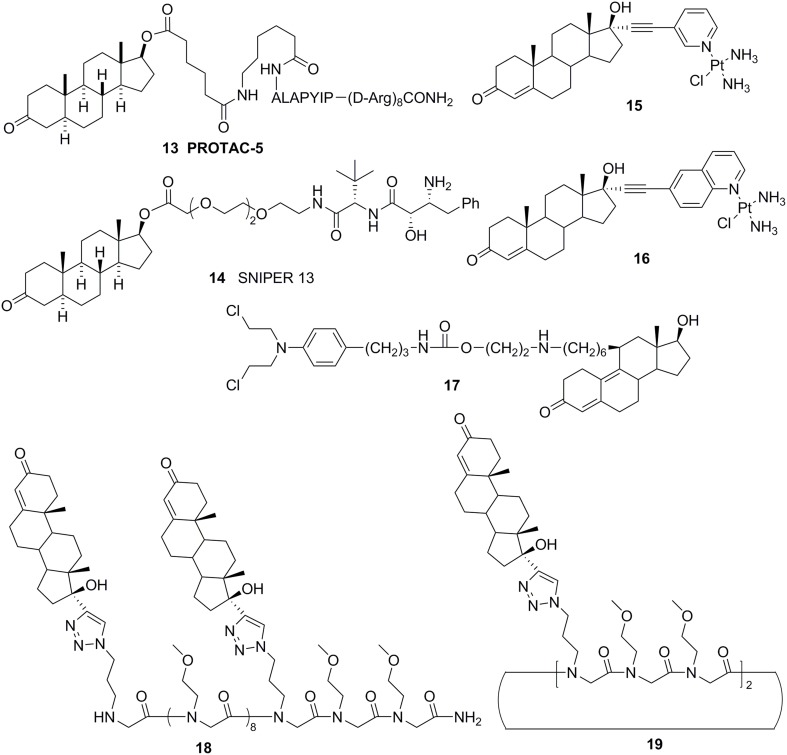
**Chemical structures of steroid conjugate AR Antagonists**.

Cross coupling and conjugation strategies are wildly applied to develop the modulator with multivalent and heterobifunctional constructs, which exhibit high affinity and specificity to the biomolecular target (Lambert, 2013; Washburn et al., 2013) (Figure 3C). To date, there are limited examples targeting the AR with steroidal conjugates (Levine et al., 2014). Utilizing conjugates dubbed PROteolysis TArgeting Chimeric moleculeS (PROTACS), the first steroid conjugate PROTAC-5 (Figure 6) to selectively induce AR degradation was developed, which consist of three components: a targeting moiety (DHT), a linker, and a recognition element for E3. Initial *ex vivo* studies showed that PROTAC-5 successfully degraded AR without compromising normal cell viability at a concentration of 25 μ M 9 (Schneekloth et al., 2004). In addition, Hashimoto lab has developed Specific and Nongenetic IAPs-dependent Protein ERasers (SNIPERs) that consist of a targeting moiety (DHT), linker, and a recognition element for IAPs. In human mammary tumor (MCF-7) cells that express AR, SNIPER 13 (Figure 6), an AR targeting compound, decreased AR protein levels at a concentration of 30 μ M. Recent studies from the Hannon group have discovered the first metallo-based chemotherapeutic conjugates targeting AR. Ethisterone was conjugated to pyridines, quinolines, and isoquinolines utilizing Sonogashira cross-coupling conditions. Subsequent coordination to platinum (II) complexes yielded metallo-based bifunctional agents. Initial evaluation of the cytotoxic effects of the two most promising metallo-based bifunctional agents **15** and **16** (Figure 6), in the cell lines that express AR revealed promising biological activity (IC_50_ = 15.9 μ M) (Huxley et al., 2010). Essigmann's group has developed heterobifunctional DNA-damaging agent **17** (Figure 6) in which a alkylating agent *N,N*-bis-2-chloroethylaniline was linked to a steroid hormone that targets AR, allowing the conjugate to simultaneously bind to AR and DNA, resulting in the blockade of DNA repair enzymes in PCa cell lines that overexpressing AR, subsequently leading to the disruption of AR-mediated transcription and signaling (Marquis et al., 2005). An emerging avenue in molecular pharmacology is the development of multivalent therapeutic agents. Therefore, the Kirshenbaum lab designed multivalent ethisterone conjugates to specifically target the AR LDB and modulate AR activity via different mechanisms of action. Ethisterone was conjugated at the 17-α position to the peptoid scaffold via highly stable triazole linakges. Two conjugates **18** and **19** (Figure 6) exhibited potent anti-proliferative properties in proliferation studies of LNCaP-abl cell lines and no cytotoxicity in PC-3 and HEK293 cell lines, establishing that conjugates selectively target AR (Levine et al., 2012).

**Figure 7:**
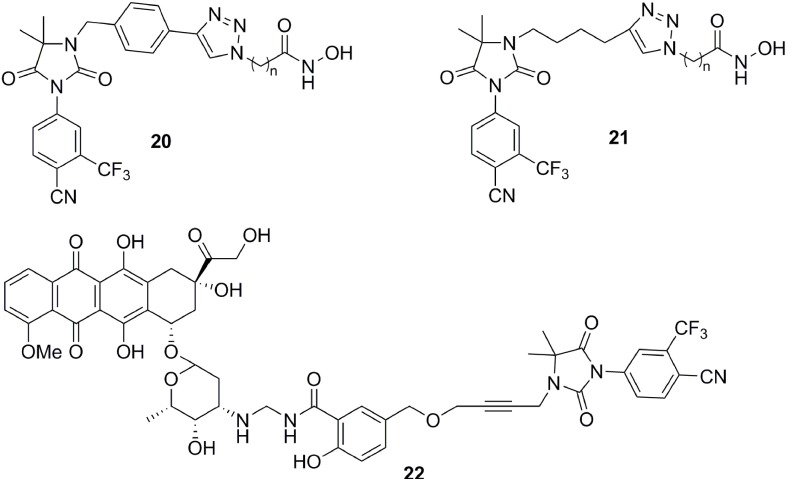
**Chemical structures of non-steroid conjugate AR Antagonists**.

Except for steroid conjugates, there are several representative examples of promising strategies that have been used to target AR with non-steroidal conjugates. Recently, the Oyelere's lab reported a non-steroidal heterobifunctional conjugate **20** (Figure 7) outfitted with histone deacetylase inhibitors that exhibit higher potency in modulation of AR activity than clinically used anti-androgens (Gryder et al., 2013). In similar studies, the Koch lab reported a non-steroidal heterobifunctional conjugate **21** (Figure 7) containing doxorubicin, a non-selective cytotoxic therapeutic DNA intercalator. The antiandrogen conjugate successfully delivered the doxorubicin-formaldehyde Schiff base to cells overexpressing AR (Cogan and Koch, 2003). The El-Sayed lab introduced the first non-steroidal multivalent conjugate **22** (Figure 7) that selectively target membrane-associated AR. Bicalutamide was conjugated to gold nanoparticles. The multivalent compounds enhanced potency by one order of magnitude, in comparison to the monovalent ligand, in PCa cells (Dreaden et al., 2012).

## Challenges and future perspectives

Despite the great improvements h ave been made in the development of antiandrogen circumventing mutation-based resistance, it remains a big challenge. First of all, there is still no antagonistic or apo AR LBD structure available, current LBD actually are all agonistic, which add the hurdle of structure-based antiandrogen design. Up to now, the adopted antagonistic AR LBD model included the homology model based on the antagonistic LBD of other nuclear receptor such as ER, GR and PR, the model with the H12 simply deleted from the agonistic AR LBD, and the model equilibrated via molecular dynamics from the started structure of the agonistic AR LBD. Thus, the antagonistic or apo AR LBD structure is highly in demand to provide more accurate model in structural based drug design of antiandogens. However, as the antagonistic or apo AR LBD protein is formidable to be prepared and crystallized due to its flexibility and tight association with the bacterial chaperonin (Bohl et al., 2005b), to solve the 3D structure of the antagonistic or apo AR LBD is still a huge challenge. Secondly, besides of acquired the mutation of AR to induce drug resistance, there are other mechanisms including aberrant expression of AR co-regulators and alternative AR activation by cytokines and growth factors, and the expression of AR splice variants lacking LBD. As an example, the cells with AR gene rearrangements expressing both full-length and AR-Vs are identified androgen independent and enzalutamide resistant, and selective knock-down of AR-Vs expression inhibited androgen-independent growth and restored responsiveness to androgens and antiandrogens (Li et al., 2013b). Moreover, it was proposed that the resistance to enzalutamide via the activation of AR and its splice variants may be mediated by NF-kB2/p52 (Nadiminty et al., 2013). Recent work revealed the enzalutamide resistance could also be achieved through the activation of GR signaling (Arora et al., 2013). Therefore, the drug resistance is associated with multiple factors, and still a long trudge is left to circumvent the antiandrogen resistance in prostate cancer.

Besides of HBP, several other binding sites on AR like AF2, BF3, the DNA binding site, and AF1 have been attracted attentions in novel antiandrogen development (Haendler and Cleve, 2012). To date, several ligands have been identified to bind to these sites, and exhibiting potent activities in antagonizing the AR signaling, which inhibit the proliferation of AR dependent prostate cancer cells (Andersen et al., 2010; Axerio-Cilies et al., 2011; Ravindranathan et al., 2013; Li et al., 2014; Munuganti et al., 2014). Some of these active agents demonstrated significant antiandrogen potency against enzalutamide-resistant prostate cancer cell lines (Li et al., 2014; Munuganti et al., 2014). Especially, the agents that target the DNA binding site or AF1 could effectively inhibit the growth of enzalutamide-resistant cells as well as block the transcriptional activity of constitutively active AR splice variants like AR-V7, ARv567 (Li et al., 2014). Thus, targeting these sites other than HBP provides an another feasible avenue to develop the therapeutic agents for prostate cancer. As it has been beyond the scope of current review, several comprehensive reviews are recommended here (Nyronen and Soderholm, 2010; Haendler and Cleve, 2012; Lallous et al., 2013; Culig, 2014; Tan et al., 2015; Lorente et al., 2015).

### Conflict of interest statement

The authors declare that the research was conducted in the absence of any commercial or financial relationships that could be construed as a potential conflict of interest.
