# Corrigendum: Progress in antiandrogen design targeting hormone binding pocket to circumvent mutation based resistance

**DOI:** 10.3389/fphar.2015.00097

**Published:** 2015-05-08

**Authors:** Xiaohong Tian, Yang He, Jinming Zhou

**Affiliations:** ^1^Lady Davis Institute, Jewish General Hospital, Mcgill UniversityMontreal, QC, Canada; ^2^Institute of Medicinal Biotechnology, Chinese Academy of Medical ScienceBeijing, China

**Keywords:** androgen receptor, antiandrogen, drug resistance, mutation, rational drug design

In the version of this article initially published online, there are two typos in Figure 3B, the “Atiandrogen design” should be “Antiandrogen design” and “ZINK” should be “ZINC.” The correction has no impact on the results of the study or its conclusions. The corrected figure is given here.

**Figure 3:**
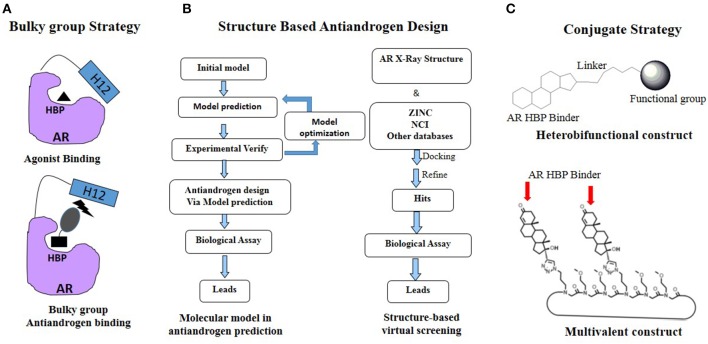
**Rational antiandrogen design strategy to combat the mutation driven drug resistance. (A)** Bulky group strategy. **(B)** Structure based antiandrogen design. **(C)** Conjugate strategy.

## Conflict of interest statement

The authors declare that the research was conducted in the absence of any commercial or financial relationships that could be construed as a potential conflict of interest.

